# GPR120 is an important inflammatory regulator in the development of osteoarthritis

**DOI:** 10.1186/s13075-018-1660-6

**Published:** 2018-08-03

**Authors:** Yuanfeng Chen, Dan Zhang, Ki Wai Ho, Sien Lin, Wade Chun-Wai Suen, Huantian Zhang, Zhengang Zha, Gang Li, Po Sing Leung

**Affiliations:** 1 Institute of Orthopedic Diseases and Center for Joint Surgery and Sports Medicine, the First Affiliated Hospital, Jinan University, Guangzhou, People’s Republic of China; 2 School of Biomedical Sciences, Faculty of Medicine, The Chinese University of Hong Kong, Hong Kong, Hong Kong SAR People’s Republic of China; 3 Department of Orthopaedics & Traumatology, Li Ka Shing Institute of Health Sciences and Lui Che Woo Institute of Innovative Medicine, Faculty of Medicine, The Chinese University of Hong Kong, Prince of Wales Hospital, Shatin, Hong Kong SAR People’s Republic of China; 4 The CUHK-ACC Space Medicine Centre on Health Maintenance of Musculoskeletal System, The Chinese University of Hong Kong Shenzhen Research Institute, Shenzhen, People’s Republic of China; 5 Department of Haematology, University of Cambridge, Cambridge, CB2 0PT UK

**Keywords:** G-protein coupled receptors, Polyunsaturated fatty acids, Proinflammatory mediators, Cartilage, Subchondral bone, Skin defect, Diagnostic markers

## Abstract

**Background:**

The aim of this study was to investigate the regulatory role of G-protein coupled receptor 120 (GPR120) in the development and progression of osteoarthritis (OA).

**Methods:**

GPR120 knockout (KO) and wild-type (WT) mice were used to create an animal model of OA by means of anterior cruciate ligament transection (ACLT) surgery**.** The severity of OA was staged and evaluated by histological examination, microcomputed tomography scan and enzyme-linked immunosorbent assay (ELISA). The anti-inflammatory effects of the GPR120 agonist docosahexaenoic acid (DHA) on human chondrocytes were further evaluated by specific inflammatory markers. In addition, the healing progression of a skin defect model was determined with histological assays.

**Results:**

The GPR120-KO mice displayed an accelerated development of OA after ACLT. The secondary inflammation, cartilage degeneration, and subchondral bone aberrant changes were significantly elevated in the early phase of OA in KO mice relative to those in WT mice. In addition, we found that GPR120 levels were downregulated in OA patients compared with control subjects, whereas GPR120 activation with DHA exhibited anti-inflammatory effects in primary human chondrocytes in vitro. Moreover, results from the skin defect model showed that GPR120 agonism with DHA enhanced wound repair in mice, as shown by the downregulation of the number of CD68^+^ cells.

**Conclusions:**

Our study suggests that GPR120 is an important inflammatory mediator during the development of OA, and that it is a potential marker for the diagnosis of high-risk patients with OA.

**Electronic supplementary material:**

The online version of this article (10.1186/s13075-018-1660-6) contains supplementary material, which is available to authorized users.

## Background

Osteoarthritis (OA) is one of the leading causes of physical disability and affects nearly 80% of individuals older than 75 years in the US [1]. Current pharmacological therapies are mainly targeted at the level of symptomatic control, which is less effective for disease progression. Better understanding of the pathogenesis of OA is crucial for the design and development of novel therapeutic agents. Obesity is one of the primary risk factors for OA, but the underlying mechanisms involved have yet to be determined [2]. It is believed that an increased loading by weight gain on the joints is attributable to the obesity-accelerated OA; however, the mechanical factors alone do not account for the higher incidence of OA in nonweight-bearing joints, such as the hands [3]. Interestingly, previous studies have shown that morbidly obese mice do not develop OA when fed with standard or low-fat diet [4]. These findings suggest that other factors rather than adiposity or body weight contribute to OA in obesity, such as lipid metabolism homeostasis or the circulating levels of adipokines.

It was reported that obesity-associated oxidative stress induces lipolysis of adipocytes and thus increases the circulating levels of free fatty acids (FAs) [5] . The circulating FAs can serve as either proinflammatory or anti-inflammatory molecules for metabolic signaling; for example, the saturated FAs can activate macrophages to secrete tumor necrosis factor (TNF)-α and interleukin (IL)-1, thereby activating the proinflammatory pathways [5]. In this regard, the derivatives of ω-6 polyunsaturated FAs (PUFA) are involved in joint pain [6, 7], while ω-3 FAs are reported to reduce spontaneous OA in animals on a low-fat diet [8]. Generally, by binding its receptor, ω-3 PUFA gives rise to anti-inflammatory oxylipins such as protectins and resolvins, whereas ω-6 PUFA produce proinflammatory oxylipins including numerous prostaglandins and leukotrienes [9]. Furthermore, it has been reported that the surface of cartilage is covered with a layer of phospholipids that serves as a boundary lubricant during joint loading [10]. Therefore, changes in the composition of this lubrication layer due to either injury or abnormal lipid metabolism may impact the function of the articular joint and potentially lead to the onset of OA [11]. These findings imply that free FAs or metabolic factors play a relatively direct role in the process of joint degeneration, but the regulatory roles of the ω-3 FAs and their receptors in the development of OA still need to be further investigated.

G-protein coupled receptor 120 (GPR120), or free fatty acid receptor 4 (FFA4), is known to bind with ω-3 and stabilize the metabolic homeostasis via cascades of physiological activities [12, 13]. Activation of GPR120 with its agonists such as docosahexaenoic acid (DHA) has an insulinotropic effect on pancreatic beta-cell secretion and survival, with therapeutic potential for obesity-associated type 2 diabetes [14]. In fact, GPR120 stimulation confers protection from obesity and diabetes by inhibiting inflammatory responses, modulating hormone secretions from the gastrointestinal tract and pancreas, and regulating lipid and/or glucose metabolism in adipose, liver, and muscle tissues [15]. However, whether GPR120 plays a role in OA is still largely unknown. The objective of this study was to investigate the role of GPR120 in the development of OA and whether it can be a potential marker for the diagnosis of high-risk patients with OA.

## Methods

### GPR120 knockout mice

GPR120 global knockout (KO) mice (Ffar4^tm1(KOMP)Vlcg^, http://velocigene.com/komp/detail/15078) were produced by the Knockout Mice Project (KOMP) Repository (UCSD, CA, USA) as has been reported previously [16]. Briefly, the targeting vector was constructed by ligating the fragments of 5’ and 3’ homology recombination arms and the fragment for the *lacZ-*ployA-loxP-hUbCpro-*neo*^*r*^-ployA-loxP cassette. The targeting vector was introduced into C57BL/6 embryonic stem cells, where the original DNA was replaced by homologous recombination. The coding region of mouse GPR120 consists of three exons, exons 1–3. The major parts of exon 1 and 3 and the whole of exon 2 were replaced with the aforementioned cassette. Using heterozygous GPR120 KO mouse sperm provided by KOMP, we established GPR120 KO mice by performing in-vitro fertilization. Our experimental procedures were approved by the Animal Experimental Ethics Committee of the Chinese University of Hong Kong (ref. 13/044/GRF-5).

### Genotyping

The last 3–5 mm of mouse tails were digested with 100 μl 50 mM NaOH for approximately 25 min in a water bath at 95 °C, and then centrifuged to remove cell debris. Real-time polymerase chain reaction (RT-PCR) analysis was performed using 1 μl genomic DNA to determine the expression of the tag gene Neomycinresistance (Neo^r^) and GPR120. Primers for the genotyping are listed in Additional file 1.

### Clinical sample collection

The study was approved by the Joint Chinese University of Hong Kong-New Territories East Cluster Clinical Research Ethics Committee (ethical approval code CRE-2013.248) or the First Affiliated Hospital of Guangzhou University of Chinese Medicine Clinical Research Ethics Committee (ethical approval code YJ-2015.034) and informed consent was obtained from each donor. The clinical specimens (cartilage or fat tissues) were obtained from patients with OA during total knee arthroplasty surgery (*n* = 10; seven women and three men; age 62.3 ± 4.5 years, range 45–72 years) in the Prince of Wales Hospital, Chinese University of Hong Kong. The clinical samples for the control group were collected from bone fracture patients with no previous history of OA during fracture surgery in the First Affiliated Hospital of Guangzhou University of Chinese Medicine (*n* = 9; five women and four men; age 58.8 ± 3.6 years, range 32–77 years).

### Animal models

Male GPR120-KO mice or wild-type (WT) mice, 12 weeks old and weighing 20–25 g, were used in this study (ref. 13/044/GRF-5). Animals were acclimatized to local vivarium conditions at a temperature of 24–26 °C and humidity of 70% with free access to water and a pelleted commercial diet in the mouse house under specific pathogen-free (SPF) conditions. For the OA model, WT or KO mice were used for the OA and control groups (*n* = 10). In the OA group, the right knee joint of the mice received anterior cruciate ligament and medial collateral ligament transection (ACLT) surgery as previously described [17]. In the sham-operated group (*n* = 10), only the skin of the right knee joint was resected. Samples were collected at 6 weeks after the operations. At 4 and 6 weeks postoperation, WT and KO mice from each group were randomly selected and killed for the collection of blood serum and right knee joint samples.

For the skin defect model (ref. 17–145-ITF), WT or KO mice were used in each group (*n* = 5). The dorsolateral skin of the mice was first punched using a 4-mm skin biopsy punch and the mice were then divided into docosahexaenoic acid (DHA; Cayman Chemical, USA) and control groups. In the DHA group, the mice were treated daily with 180 μl DHA (7 mg/ml) by gavage administration; in the control group, the mice were given phosphate-buffered saline (PBS) by gavage. Photos of the wound were taken for 8 consecutive days and wound sizes were estimated using ImageJ software (National Institutes of Health, Bethesda, MD.). All mice were sacrificed at day 8 and skin samples were collected.

### Cell experiments

Nonfibrillated cartilage samples (OARSI scores of 0–3) collected from patients during total knee arthroplasty surgery were analyzed in this study. The cartilage tissues were washed and minced into pieces before being sequentially digested with 0.25% trypsin (Life, USA) for 20 min and 0.2% (2 mg/ml) type II collagenase (Sigma, USA) for 24 h at 37 °C. After centrifugation, the supernatants were removed and the chondrocytes were cultured in alpha minimum essential medium (α-MEM) + 10% fetal bovine serum (FBS) (both from Invitrogen Corp., Carlsbad, CA, USA). The cell type was identified by collagen II immunostaining.

For the inflammatory induction study in the TNF-α + DHA group, chondrocyte cells at passages 2 to 3 were seeded on 24-well plates (5 × 10^4^ cells/well) in serum-free Dulbecco’s modified Eagle’s medium (DMEM) and treated with 50 ng/ml TNF-α (Sigma, USA) and 10 μg/ml DHA (Cayman Chemical, USA). For the TNF-α group, cells were treated with 50 ng/ml TNF-α; neither TNF-α nor DHA were applied to the cells in the control group. Cells were harvested after 24 h of incubation.

The gene expression levels of chemokine (C-C motif) ligand 2 (Ccl2), cyclooxygenase 2 (Cox2), IL-1β, matrix metallopeptidase (MMP)-13, and glyceraldehyde 3-phosphate dehydrogenase (GAPDH) for chondrocytes induced by TNF-α were determined using RT-PCR. Primer sequences are listed in Additional file 2.

### Enzyme-linked immunosorbent assay (ELISA) measurements

For the human clinical samples, fat tissues were collected (1 × 1 cm^3^) from the OA patient group and the non-OA patient control group. Samples were weighed, mechanically homogenized and ground into powder with liquid nitrogen, and then were treated with ice-cold tissue protein extraction reagents (Life Technologies, Pleasanton, CA, USA). Samples were then centrifuged and tested using a GPR120 ELISA examination kit according to the protocol suggested by the manufacturer (Fine Test, China).

For the animal samples from the OA model, a 1-ml blood sample was collected by cardiac puncture immediately after the mice were sacrificed. The blood sample was then centrifuged and the TNF-α level tested using a TNF-α ELISA kit according to the protocol suggested by the manufacturer (Dakewe, China).

### Microcomputed tomography (μCT) assessment

The right knee joints of mice from the OA model were fixed overnight in 10% formalin. Samples were then analyzed using high-resolution μCT scan (μCT40, Scanco Medical, Basserdorf, Switzerland). Three dimensional (3D) reconstructions of the mineralized tissues were performed using a global threshold (216 mg hydroxyapatite/cm^3^) and a Gaussian filter (sigma = 0.8, support = 2) was used for noise suppression. One hundred sagittal images of the tibial subchondral bone were used to perform the 3D histomorphometric analysis. The bone mineral density (BMD), bone volume/total tissue volume (BV/TV), trabecular thickness (Tb.Th) and structure model index (SMI) were analyzed as the 3D structural parameters.

### Histological and immunochemical examinations

Mouse tissue samples including colon tissues from GPR120 KO mice, right knee joints collected from the OA model, the skin of the back from the skin defect model, and the human chondrocytes were fixed in 10% formalin, while the knee joint was additionally treated with 10% ethylenediaminetetraacetic acid (EDTA) for decalcification for 14 days before paraffin embedding. Frozen samples were embedded in the optimum cutting temperature (OCT) compound (Sakura Finetek, Zoeterwoude, The Netherlands) and then sectioned at 5 μm thick for skin and right knee joint samples and 6 μm thick for colon samples at the sagittal-oriented position for Safranin-O/fast green, hematoxylin and eosin (H&E), and immunofluorescent staining.

For immunofluorescent staining, the colon tissues and human chondrocyte were incubated with primary antibodies to chicken anti-beta galactosidase (Abcam, 1:100, ab9361) and rabbit anti-Collagen II (Abcam, 1:300, ab34712), respectively, overnight at 4 °C. Secondary antibodies conjugated with fluorescence goat anti-chicken or goat anti-rabbit CY3 (Life Technologies, Pleasanton, CA, USA; 1:800) were added, and slides were incubated at room temperature in the dark for 1 h. Photographs of the selected areas were taken under a microscope.

Immunostaining was performed using a standard protocol as previously reported [18, 19]. We incubated the sections with primary antibodies to rabbit MMP13 (Abcam, 1:50, ab3208) and collagen X (Abcam, 1:80, ab58632), Osterix (Abcam, 1:600, ab22552), and CD68 (Boster, 1:100, BA2966) overnight at 4 °C. For immunohistochemical staining, a horseradish peroxidase–streptavidin in the detection system (Dako, Carpinteria, CA, USA) was subsequently used, followed by counterstaining with hematoxylin. Photographs of the selected areas were taken under a light microscope. We counted the number of positively stained cells and repeated in triplicate in three randomly selected sections in the area of interest per specimen, and the numbers of cells were statistically analyzed.

### Statistical analysis

In accordance with the ARRIVE guidelines [20], we have reported measures of precision and *n* to provide an indication of significance. All statistical analyses were performed using the statistical software SPSS 15.0. The data were analyzed by one-way analysis of variance (ANOVA), except for data on wound healing analysis which were tested by two-way ANOVA. Data are reported as mean and 95% confidence interval (CI). The graphs were generated using GraphPad Prism 6 (GraphPad Software, San Diego, CA, USA).

## Results

### Validation of GPR120-KO mice

**Fig. 1:**
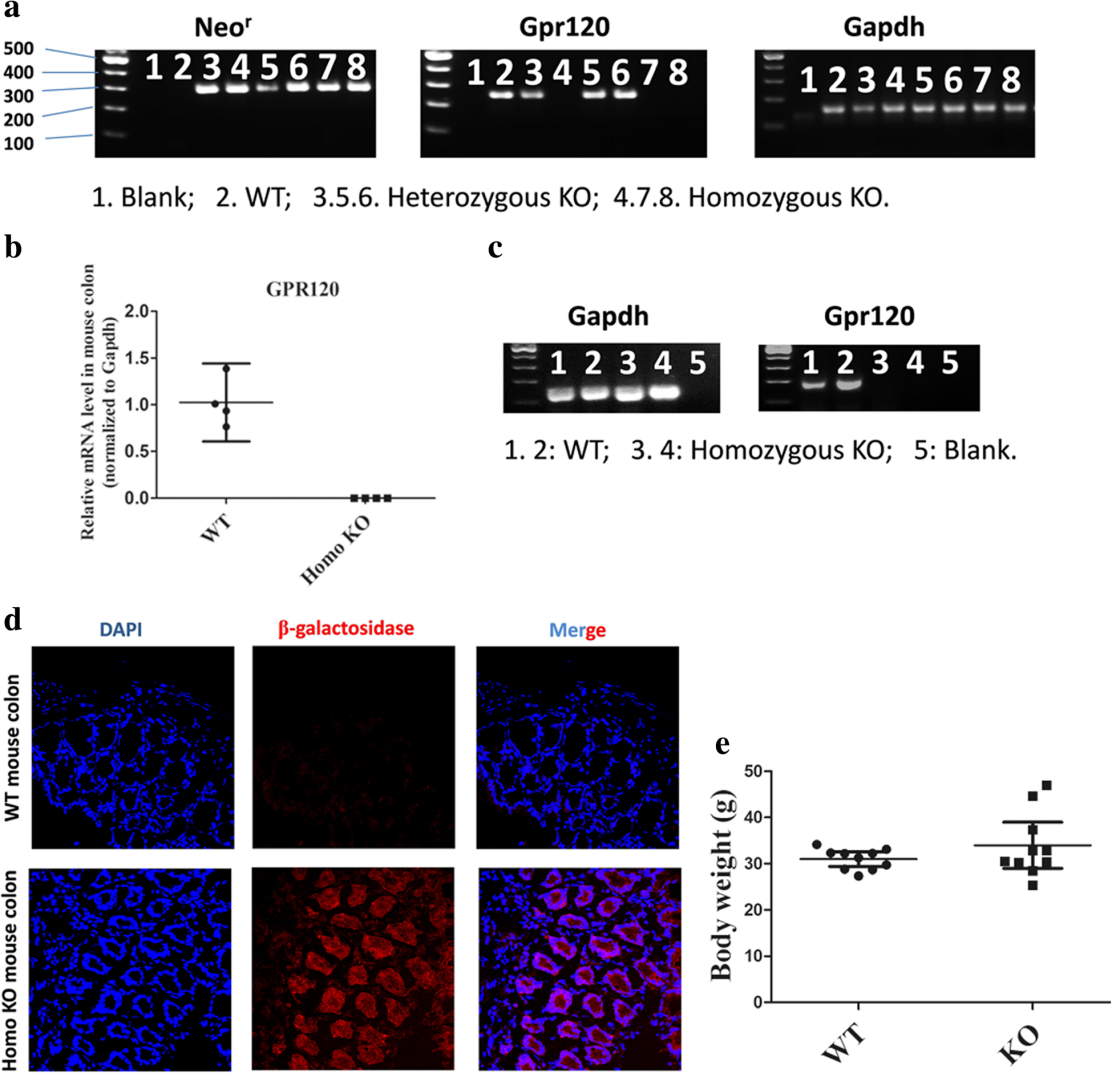
Validation of GPR120 knockout mice. **a** Agarose gels demonstrate Neo^r^, Gpr120, and Gapdh amplification products in mouse genomic DNA. **b** The GPR120 mRNA level was detected by real-time PCR in colon (positive control tissue) of wild-type (WT) and homozygous knockout (Homo KO) mice (*n* = 4/group). **c** Agarose gels demonstrate Gapdh and Gpr120 amplification products in mouse colon cDNA. **d** The presence of β-galactosidase, a surrogate for GPR120 in the KO mouse, is detected by immunofluorescence in mouse colon tissue. Colon sections from WT (upper) and homo KO (bottom) mice were stained with antibody against beta-galactosidase (red). Magnification of the image ×100. **e** The body weight of the two group do not show statistical difference

Genotyping result showed that only WT mice showed expression of GPR120 at the genomic DNA (Fig. 1a) or mRNA level (Fig. 1b, c), while such expression was not detected in GPR120-KO mice. β-galactosidase activity is a surrogate of GPR120, and immunofluorescent staining also showed that β-galactosidase-positive cells (red) could be found in colon tissue of the KO mice while it was absent in WT mice (Fig. 1d). These results indicated that knockout of the GPR120 gene was successfully generated. In addition, changes in body weight of the KO and the WT mice were also found to be not statistically different (mean 33.97, 95% CI 28.97–38.98 g, versus 30.99, 95% CI 29.41–32.56 g, respectively; *n* = 10; *p* > 0.05) (Fig. 1e). Taken together, these results provide evidence of the successful establishment of the GPR120 KO mice.

### Acceleration of cartilage degeneration in GPR120-KO mice with surgically induced OA

**Fig. 2:**
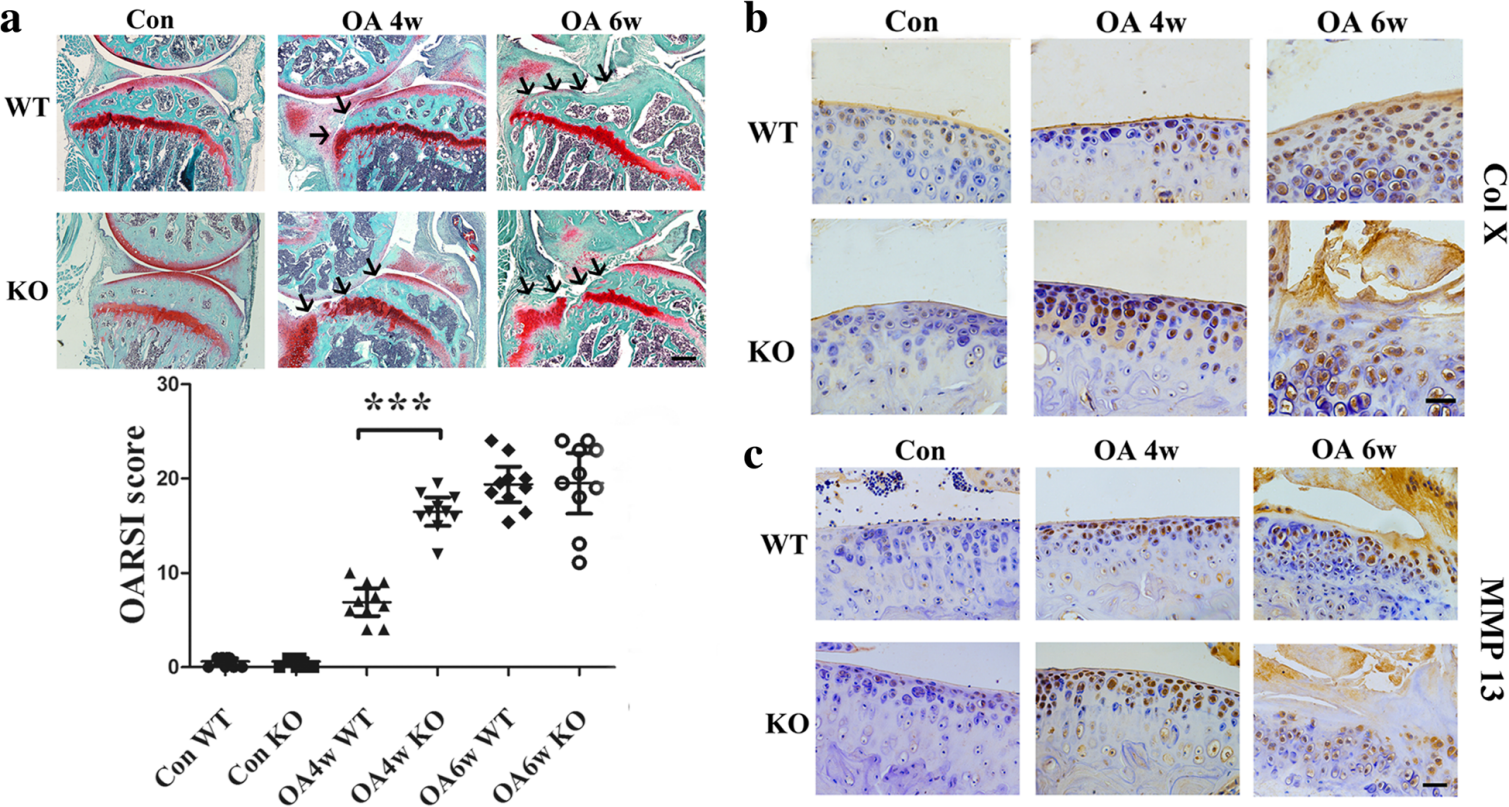
**a** Safranin-O/fast green staining and quantification of the histologic results using the Osteoarthritis Research Society International Cartilage Histopathology Assessment System (OARSI score) indicated articular cartilage damage in all groups. Black arrows show the damaged region of the cartilage. **p < 0.01, compared with the wild-type (WT) osteoarthritis (OA) mice at 4 weeks (4w). Scale bar = 400 μm. Immunohistochemical analysis of **b** type X collagen (COL X)- and **c** matrix metalloproteinase 13 (MMP13)-positive chondrocytes (brown) in articular cartilage showed that GPR120 knockout (KO) mice significantly increased the numbers of COL X- and MMP13-positive chondrocytes compared with the WT mice 4 weeks after the OA surgery. The fewest numbers of positive cells could be found in the sham control (Con) in both KO and WT mice, and the highest numbers of COL X- and MMP13-positive cells can be found in OA at 6 weeks (6w) for both KO and WT mice. Scale bar = 50 μm

Safranin-O/fast green staining showed that there were significantly more degenerative features in the knee joint samples of KO mice compared with those of WT mice at 4 weeks postoperation, while the cartilage damage is obvious in both groups 6 weeks after the OA surgery. No abnormal cartilages were observed in the sham group (Fig. 2a). Based on the OARSI histologic grading system, the scores indicated that KO mice showed more severe cartilage degeneration (16.5, 95% CI 15.02–17.98; *n* = 10) than WT mice (6.9, 95% CI 5.441–8.358; *n* = 10; *p* = 0.0034) at 4 weeks postoperation, and the cartilage damage was significantly worse in both KO and WT mice at 6 weeks postoperation (19.52, 95% CI 16.32–22.72, and 19.38, 95% CI 17.49–21.27, respectively; *n* = 10; *p* > 0.05). For the sham group, both KO and WT mice showed minimum cartilage damage (0.6, 95% CI 0.2306–0.9694, versus 0.6, 95% CI 0.23–0.97, respectively; *n* = 10; *p* > 0.05) (Fig. 2a and Additional file 3). Moreover, the percentage of type X collagen (ColX)-positive chondrocytes and MMP13^+^ chondrocytes in cartilage were both higher in the KO mice (46.2, 95% CI 39.35–53.04, and 50.19, 95% CI 46.11–54.28, respectively; *n* = 5; *p* < 0.01) than in WT mice (32.70, 95% CI 27.66–37.74, and 32.92, 95% CI 26.73–39.11, respectively; *n* = 5) at 4 weeks after surgery (Fig. 2b, c and Additional file 4A, B). In the sham control group, both KO and WT mice showed the lowest percentage of ColX^+^ chondrocytes (KO: 25.96, 95% CI 19.22–32.70; WT: 27.51, 95% CI 23.19–31.82; *n* = 5) and MMP13^+^ chondrocytes (KO: 23.88, 95% CI 16.66–31.09; WT: 25.95, 95% CI 17.34–34.56; *n* = 5). The highest percentage of ColX^+^ (KO: 57.46, 95% CI 51.75–63.17; WT: 52.86, 95% CI 46.93–58.79; *n* = 5) and MMP13^+^ chondrocytes (KO: 56.82, 95% CI 52.23–61.41; WT: 52.14, 95% CI 46.86–57.41; *n* = 5) were found in both KO and WT mice at 6 weeks postoperation (Fig. 2b, c and Additional file 4A, B).

### Aggravation of abnormal bone remodeling in subchondral bone in GPR120-KO mice with surgically induced OA

**Fig. 3:**
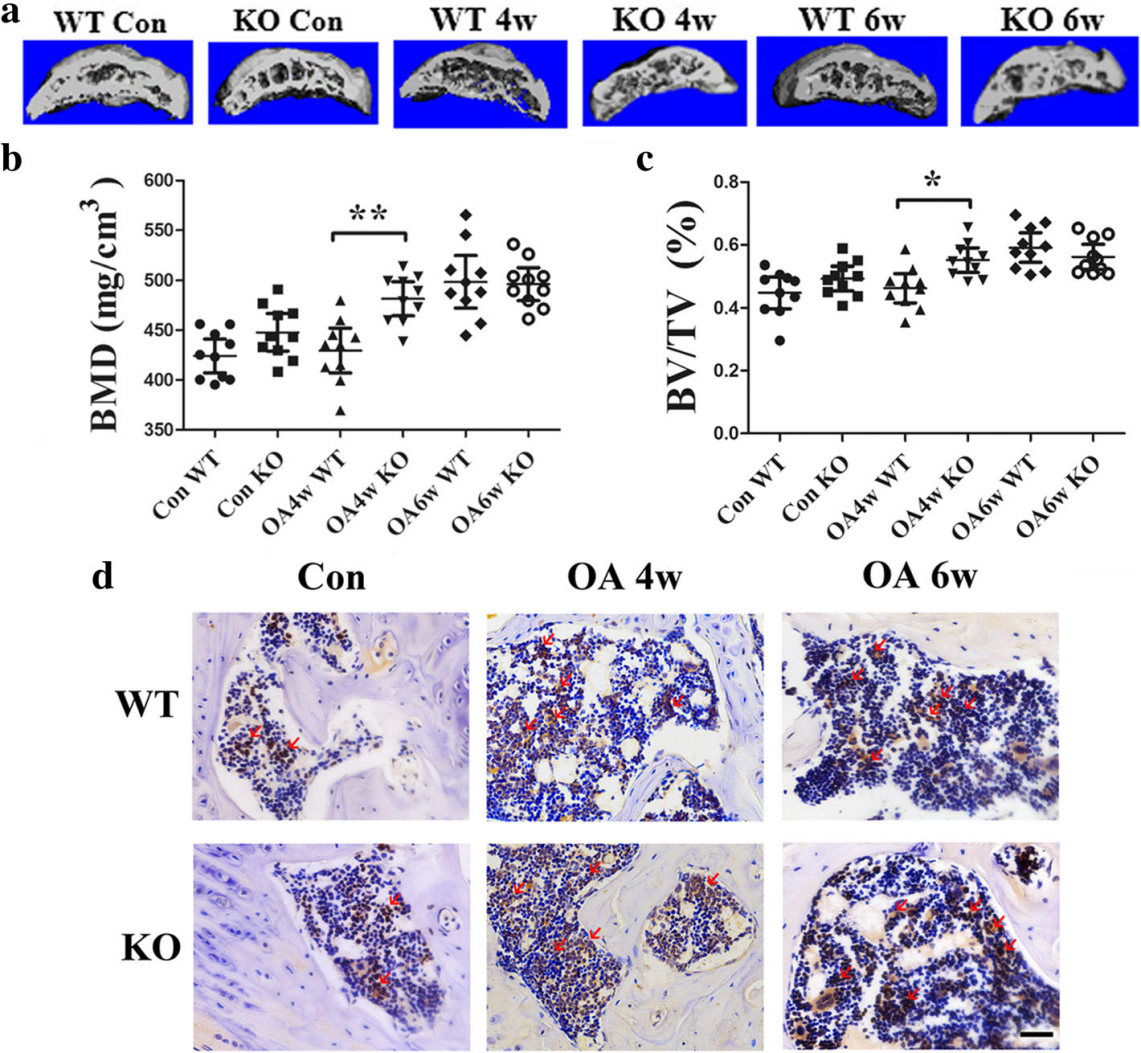
**a** 3D μCT images of the tibia subchondral bone medial compartment (sagittal view) of mice in all groups. Quantitative analysis of structural parameters of subchondral bone by μCT. **b** Bone mineral density (BMD) and **c** bone volume/total tissue volume (BV/TV), *n* = 10 per group. *p < 0.05, compared with the wild-type (WT) osteoarthritis (OA) mice at 4 weeks (4w). **d** Immunohistochemical analysis of Osterix-positive cells (brown, red arrow) in the tibial subchondral region. The result showed that GPR120 knockout (KO) mice significantly increased the numbers of Osterix-positive cells in subchondral bone compared with the WT mice 4 week after OA surgery. Scale bar = 50 μm. 6w 6 weeks, Con sham control

The 3D reconstructed images from μCT showed the microarchitecture of the mouse subchondral bone in all groups (Fig. 3a). The results showed that, at 4 weeks after OA surgery, the abnormal bone formation in subchondral bone was significantly more severe in KO mice (BMD: 481.5, 95% CI 464.5–498.6 mg/cm^3^, *n* = 10; BV/TV: 0.5515, 95% CI 0.5130–0.5901, *n* = 10) than in WT mice (BMD: 429.5, 95% CI 406.9–452.1 mg/cm^3^, *n* = 10, *p* = 0.0256; BV/TV: 0.4630, 95% CI 0.4162–0.5097, *n* = 10, *p* = 0.0359) (Fig. 3b, c). At 6 weeks after operation, the abnormal bone formation was severe in both KO (BMD: 496.3, 95% CI 461.9–532.1 mg/cm^3^, *n* = 10; BV/TV: 0.5619, 95% CI 0.5225–0.6014, *n* = 10) and WT mice (BMD: 498.4, 95% CI 472.1–524.7 mg/cm^3^, *n* = 10, *p* = 0.7446; BV/TV: 0.5913, 95% CI 0.5441–0.6385, *n* = 10, *p* = 0.736) (Fig. 3b, c). In the sham control group, abnormal bone formation was mild in both KO (BMD: 447.9, 95% CI 429.0–466.8 mg/cm^3^, *n* = 10; BV/TV: 0.4935, 95% CI 0.4543–0.5326, *n* = 10) and WT mice (BMD: 424.2, 95% CI 407.2–441.1 mg/cm^3^, *n* = 10, *p* = 0.22; BV/TV: 0.4482, 95% CI 0.3971–0.4992, *n* = 10, *p* = 0.3957) (Fig. 3b, c).

Trabecular bone thickness (Tb.Th.) in KO mice (0.1505, 95% CI 0.1436–0.1575 mm; *n* = 10) was higher than that in WT mice (0.1292, 95% CI 0.1219–0.1364 mm; *n* = 10; *p* = 0.0217) at 4 weeks postoperation, and at 6 weeks after surgery Tb.Th was at the highest level for both WT and KO mice (0.155, 95% CI 0.1438–0.1662 mm, and 0.1676, 95% CI 0.1616–0.1736 mm, respectively; *n* = 10; *p* > 0.05) (Additional file 4D). Tb.Th. was lowest in KO and WT mice from the sham group (0.1208, 95% CI 0.1145–0.1272 mm, and 0.1108, 95% CI 0.1016–0.1201 mm, respectively; *n* = 10) (Additional file 4D). Furthermore, KO mice had a significantly more decreased SMI (−1.167, 95% CI −1.846 to −0.4884; *n* = 10) than WT mice (0.0036, 95% CI−0.6356 to 0.6283; *n* = 10; *p* > 0.05) at 4 weeks after the OA surgery, though no statistically significant differences were found (Additional file 4E). SMI was at the lowest level for both WT and KO mice (−1.985, 95% CI −3.073 to −0.8975, and −0.6783, 95% CI −1.351 to −0.005, respectively; *n* = 10; *p* > 0.05) at 6 weeks postoperation; in the controls, SMI in WT mice was 0.2041 (95% CI −0.1689 to 0.5772) and in KO mice it was −0.054 (95% CI −0.4845 to 0.375). There were no statistically significant differences (Additional file 4E).

Furthermore, the results of immunohistochemistry staining with Osterix, an osteoprogenitor, revealed that KO mice had a significantly higher increase in numbers of Osterix-positive cells in the subchondral bone marrow than WT mice 4 weeks after OA surgery (KO: 277.8, 95% CI 250.9–304.7; WT: 151.2, 95% CI 130.8–171.6; *n* = 5, *p* < 0.001) (Fig. 3d and Additional file 4C), while at 6 weeks postoperation an upregulated number of Osterix-positive cells could be observed in both KO and WT mice (KO: 335.2, 95% CI 293.3–377.1; WT: 323.6, 95% CI 285.3–361.9; *n* = 5). Only a few Osterix-positive cells could be found in the sham control groups for both KO and WT mice (KO: 112.6, 95% CI 87.83–137.4; WT: 115.4, 95% CI 78.30–152.5; *n* = 5) (Fig. 3d and Additional file 4C).

### Upregulation of plasma levels of TNF-α in GPR120-KO mice with surgically induced OA

**Fig. 4:**
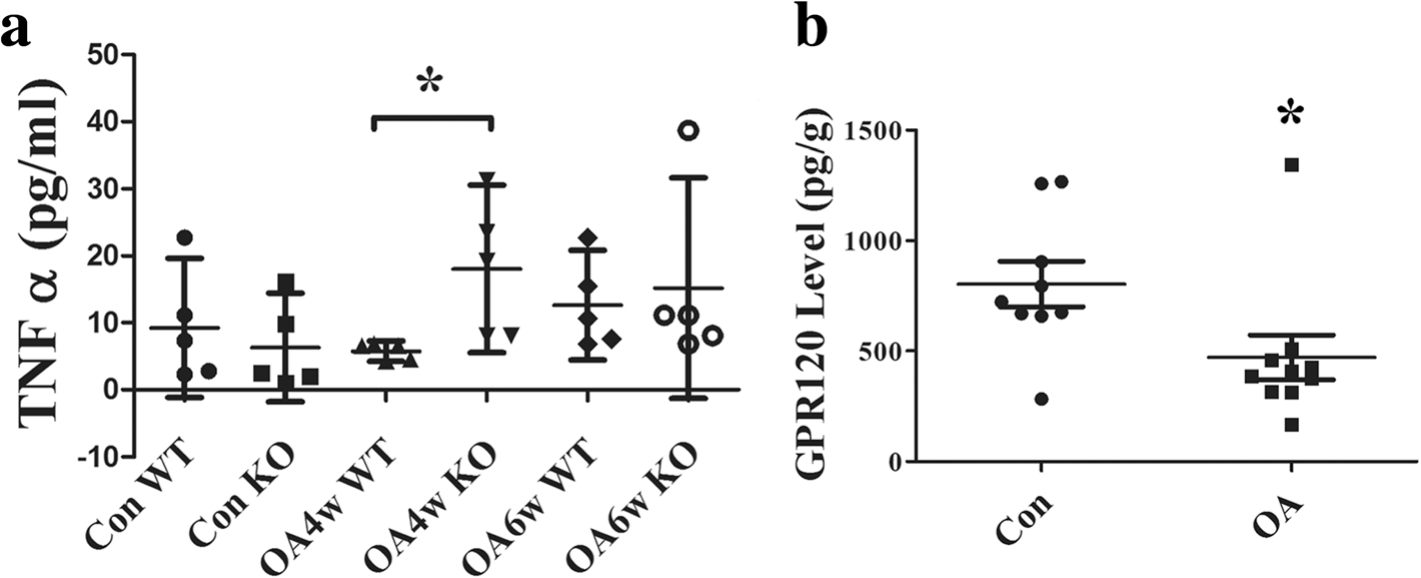
ELISA showing **a** the tumor necrosis factor alpha (TNFα) level in serum in all groups (*n* = 5 for each group; *p < 0.05, compared with the wild-type (WT) osteoarthritis (OA) mice at 4 weeks (4w)) and **b** the GPR120 level in OA (*n* = 10) and non-OA patients (*n* = 9). *p < 0.05, compared with sham control (Con). 6w 6 weeks

ELISA showed that the level of TNF-α was significantly higher in KO mice (18.04, 95% CI 5.08–30.56 pg/ml; *n* = 5) when compared with that of WT mice (5.54, 95% CI 3.436–7.645 pg/ml; *n* = 5; *p* = 0.022) at 4 weeks after the OA surgery (Fig. 4a). At 6 weeks after the operation, TNF-α was at a high level in both KO (15.15, 95% CI −1.347 to 31.65 pg/ml; *n* = 5) and WT mice (12.62, 95% CI 4.442–20.81 pg/ml; *n* = 5). The sham control group showed the lowest TNF-α level in both KO (6.3, 95% CI −1.820 to 14.42 pg/ml; *n* = 5) and WT mice (9.234, 95% CI −1.162 to 19.63 pg/ml; *n* = 5) (Fig. 4a).

### Downregulation of GPR120 expression in OA patients

ELISA was used to analyze the human clinical samples collected from the OA or non-OA patients during surgery. The result indicated that the GPR120 level was significantly more downregulated in OA patients (470.5, 95% CI 368.9–572.1 pg/ml; *n* = 10) than non-OA patients (803.6, 95% CI 700.2–907 pg/ml; *n* = 9; *p* = 0.0349) (Fig. 4b).

### GPR120 activation-induced inhibition of inflammatory factor expression in human chondrocytes

**Fig. 5:**
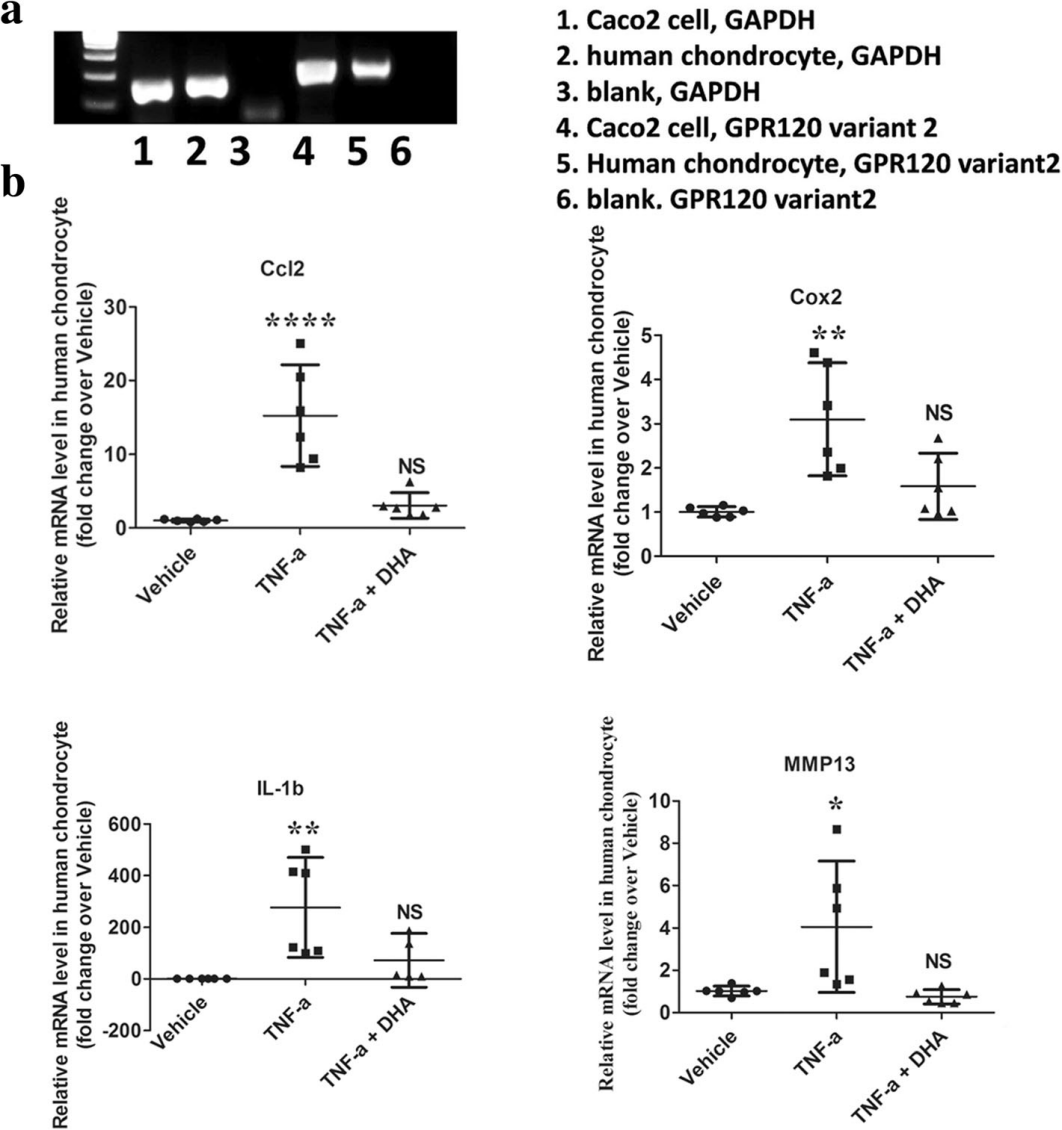
The GPR120 agonist DHA exhibits anti-inflammatory effects on primary human chondrocytes. **a** Agarose gels demonstrate Gapdh and Gpr120 amplification products in human colorectal tumor cell Caco2 (positive control) and human chondrocyte cell cDNA. **b** Human chondrocytes were exposed to 50 ng/ml human tumor necrosis factor alpha (TNFα) with or without 25 μM docosahexaenoic acid (DHA) for 48 h. The mRNA expression levels of the proinflammatory genes Ccl2, Cox2, IL-1β, and MMP13 were assessed (*n* = 6/group). *p < 0.05, **p < 0.01, ****p < 0.0001 compared with corresponding vehicle group. NS not significant

The result of immunofluorescent staining showed that the primary human chondrocytes expressed Collagen II (a chondrocyte marker) (Additional file 5). It has been reported that the chondrocytes express GPR120 [21] and, in this study, the RT-PCR result showed that DHA, a GPR120 agonist, could induce anti-inflammatory effects in primary human chondrocytes. The mRNA expression levels of the proinflammatory genes Ccl2, Cox2, and IL-1β in the TNF-α + DHA group (3.05, 95% CI 1.305–4.79; 1.58, 95% CI 0.8351–2.334; 72.93, 95% CI −32.57 to 176.7; respectively; *n* = 6) were significantly more reduced than those in the TNF-α group (15.26, 95% CI 8.374–22.14; 3.1, 95% CI 1.821–4.378; 277.01, 95% CI 83.63–470.4; respectively; *n* = 6; *p* < 0.05) (Fig. 5). Consistent with this, the TNF-α + DHA group (0.75, 95% CI 0.415–1.087; *n* = 6) had more dramatically downregulated MMP13 mRNA levels than the TNF-α group (4.05, 95% CI 0.9548–7.153; *n* = 6; *p* < 0.05) (Fig. 5).

### GPR120 activation-induced protective effect on wound repair in mice

The DHA-treated GPR120 mice showed significantly improved tissue regeneration with thickened epithelium (Additional file 6A). All mice demonstrated a certain degree of regeneration, including re-epithelialization and new formation of sebaceous glands or hair follicles, while only the DHA-treated mice showed accelerated wound repair (Additional file 6B). The infiltration of inflammatory cells to the wound margins was evaluated using the macrophage marker CD68, and the number of CD68-positive cells significantly decreased in the DHA-treated mice (146.5, 95% CI 119.1–174; *n* = 5) compare with the control mice (63.47, 95% CI 38.84–88.09; *n* = 5; *p* < 0.001) (Additional file 6C, D).

## Discussion

This study demonstrated that GPR120 is an important inflammatory regulator in the development of OA and wound healing. Several studies have shown that ω-3 PUFA bind to its receptor GPR120 to give rise to anti-inflammatory oxylipins such as protectins and resolvins, whereas ω-6 PUFA produce proinflammatory oxylipins including numerous prostaglandins and leukotrienes [9]. Additionally, it has been reported that the surface of cartilage is covered by a layer of phospholipids [10]. Any compositional changes in this lubrication layer due to either injury or abnormal lipid metabolism may have an impact on the function of the articular joint and potentially lead to the onset of OA [11]. Recently, several studies have demonstrated that lipid metabolic homeostasis plays an important role in cartilage degeneration during the development of OA [22, 23]. In our study, the results were consistent with the previous studies, showing that cartilage degeneration was significantly increased in the early phase of OA (i.e., 4 weeks postoperation) under the GPR120-KO condition; these observations were shown by the increases in the OARSI score and the expression of MMP13 and COLX (Fig. 2 and Additional files 3 and 4), as demonstrated by Safranin O/fast green and immunohistochemical staining, relative to the changes in WT mice.

Changes in subchondral bone play a key role in the regulation of OA progression [24]. In addition, bone marrow lesions are closely associated with pain, which has been implicated to predict the severity of cartilage damage in OA [25]. In-vitro studies have previously reported that GPR120 signaling negatively regulates osteoclast differentiation, survival, and function [26]. Moreover, it has also been shown that GPR120 activation-mediated cellular signaling determines the bi-potential of osteogenic and adipogenic differentiation of bone marrow-derived mesenchymal stem cells (BMSCs) in a dose-dependent manner [27]. Given that BMSCs and osteoclasts play a pivotal role in bone remodeling of the subchondral bone, these prior study findings point to the activation of GPR120 signaling as being of physiological importance for bone homeostasis. In fact, several in-vivo studies have shown that downregulation of the ω-3-GPR120 signaling leads to abnormalities in bone remodeling or osteophyte formation of subchondral bone in animal model of OA [22, 23]. Interestingly, we also found that subchondral bone aberrant changes in GPR120-KO mice were specifically increased in the early phase of OA (at 4 weeks postoperation) when compared with WT mice in the present study (Fig. 3 and Additional file 4).

It is known that inflammatory cytokines are the key regulators of OA [19, 28–30], while the activation of FA signaling has a critical role in the regulation of anti-inflammation during OA and wound healing. For example, activation of GPR120 with ω-3 PUFA is negatively correlated with the severity of OA and the area of wound healing, whereas it is positively correlated with adiponectin, an adipokine capable of promoting insulin sensitization and priming macrophages toward the M2 anti-inflammatory phenotype [23, 31, 32]. More interestingly, it has been shown that infiltration of macrophages within human adipose tissue significantly inhibits GPR120 expression [33]. In light of these findings, it is plausible to postulate that the downregulation of GPR120 expression can disrupt the lipid metabolic homeostasis, thus aggravating the level of inflammatory responses and ultimately leading to the vicious cycle in the process of OA and would healing. In corroboration with previous findings, our in-vitro studies firstly demonstrated that activation of GPR120 signaling inhibits the expression of inflammatory factors in human chondrocytes (Fig. 5). Secondly, our in-vivo studies further demonstrated that the level of secondary inflammation in GPR120-KO mice was dramatically increased in the early phase of OA (at 4 weeks postoperation) when compared with WT mice (Fig. 4a). In addition, our skin defect model indicated that GPR120 agonism with DHA could accelerate the wound repair and downregulate the inflammation level at the wound, as evidenced by the lowered number of CD68^+^ macrophages (Additional file 6). Our study findings are in agreement with a recent study reporting that the defected wound closure and cartilage regeneration may share a common, heritable, and OA-associated genetic trait [34].

One of the major obstacles to developing a new treatment option for OA is the lack of an effective and minimally invasive method to predict, diagnose, and monitor its disease progression. To address this issue, considerable efforts have been recently made for the identification of biomarkers in a clinical setting. In this regard, a lot of research for biomarkers has been focused on the release of cartilage matrix proteins, such as the collagens, proteoglycans, or cartilage oligomeric matrix protein, in the serum and synovial fluid [35–37]. Accumulated evidence has emerged that the development of OA is not only due to cartilage damage, but also to both systemic and local intra-articular metabolic factors, notably inflammation, which appear to play a pivotal role in joint degeneration [38, 39]. In additional to matrix degradation products, inflammatory cytokines and lipid metabolic factors may therefore be potential biomarkers of OA that are associated with disease mechanisms [40, 41]. Towards this end, one of the novel findings in the present study is that the expression level of GPR120 was found to be downregulated significantly more in patients with OA than in non-OA patients (Fig. 4b).

A limitation of this study is the relatively low number of mice in each group. More time points are also needed for this OA model to further evaluate the effect of GPR120 on OA progression. Also, we collected the OA and non-OA groups for the clinical specimen test without applying Kellgren grading in the assessment which might help us to understand the level of GPR120 in OA patients at different stages. Future research should consider using a larger sample size, design multiple time points in the animal study, and use Kellgren grading in the assessment to further dissect the effects of GPR120 on OA progression.

## Conclusions

In conclusion, our data indicate that GPR120 plays an important role in metabolic homeostasis by showing that GPR120 downregulation is able to interrupt the metabolic homeostasis. The dysregulated metabolism may subsequently lower the capability of immunoregulation and elicit more severe immunological reactions upon injury. In addition, the assessment of the GPR120 level may potentially be applied as a diagnostic marker for high-risk OA patients. This is the first study to report that downregulation of GPR120 is a high-risk factor for the pathogenesis of OA, and these data provide a scientific basis for the development of new minimally invasive methods (such as fat tissue biopsy) to identify the high-risk OA patients who could receive supplementation with GPR120 agonists as a potential preventative and therapeutic approach to OA.

## Additional files

Additional file 1: Primer sequence for mouse genotyping. (PDF 52 kb)

Additional file 2: Primer sequence for quantitative RT-PCR. (PDF 55 kb)

Additional file 3: Safranin O and fast green staining of sagittal sections of the subchondral tibia medial compartment in all group. Scale bar = 800 μm. (PDF 230 kb)

Additional file 4: Quantitative assay of immunohistochemical staining for positive cells and structural parameters of subchondral bone by μCT. (A) Percentages of MMP13, (B) type X collagen (COLX)-positive chondrocytes in articular cartilage, and (C) The number of Osterix-positive cells in the tibial subchondral region. (D) Tb.Th. and (E) SMI in subchondral bone determined by μCT. *n* = 5 per group. ***p < 0.001, **p < 0.01, *p < 0.05, compared with the WT OA at 4 weeks. (PDF 175 kb)

Additional file 5: Immunofluorescence staining in human chondrocytes. The result showed that human chondrocytes were type II collagen (Col II)-positive (red). Magnification of the image ×100. (PDF 93 kb)

Additional file 6: GPR120 agonist accelerates wound repair in a mouse skin defected model. (A) Representative images and quantitative assay of the wound area at 8 days post-wounding. The DHA-treated mice had the smallest wound area compared with the control mice. Two-way ANOVA, *p < 0.05. (B) H&E stained images showed that the mice treated by DHA had a thickened epithelium. The bottom figures are higher magnification views of the rectangle areas in the upper figures. Black arrow: epidermis; red arrow: hair follicle. Scale bar = 800 μm (top), 200 μm (bottom). (C) Quantitative and (D) immunohistochemical analysis of CD68-positive cells (brown) in the wound healing region. The result indicated that the number of macrophage marker (CD68)-positive cells in DHA-treated mice is downregulated significantly compared with the control mice. The bottom figures are higher magnification views of the rectangle areas in the upper figures. ***p < 0.001. Scale bar = 400 μm (top), 50 μm (bottom). (PDF 227 kb)

## Abbreviations

3D: Three-dimensional
ACLT: Anterior cruciate ligament transection
ANOVA: Analysis of variance
BMD: Bone mineral density
BMSC: Bone marrow-derived mesenchymal stem cell
BV/TV: Bone volume/total tissue volume
Ccl2: Chemokine C-C motif ligand 2
CI: Confidence interval
Col X: Type X collagen
Cox2: Cyclooxygenase 2
DHA: Docosahexaenoic acid
ELISA: Enzyme-linked immunosorbent assay
FA: Fatty acid
GAPDH: Glyceraldehyde 3-phosphate dehydrogenase
GPR120: G-protein coupled receptor 120
KO: Knockout
IL: Interleukin
MMP: Matrix metallopeptidase
μCT: Microcomputed tomography
OA: Osteoarthritis
PUFA: Polyunsaturated fatty acids
RT-PCR: Real-time polymerase chain reaction
SMI: Structure model index
Tb.Th: Trabecular thickness
TNF: Tumor necrosis factor
WT: Wild-type

## References

[1] Lawrence RC, Felson DT, Helmick CG, Arnold LM, Choi H, Deyo RA, Gabriel S, Hirsch R, Hochberg MC, Hunder GG (2008). Estimates of the prevalence of arthritis and other rheumatic conditions in the United States. Part II. Arthritis Rheum.

[2] Aspden RM (2011). Obesity punches above its weight in osteoarthritis. Nat Rev Rheumatol.

[3] Felson DT, Chaisson CE (1997). Understanding the relationship between body weight and osteoarthritis. Baillieres Clin Rheumatol.

[4] Griffin TM, Huebner JL, Kraus VB, Guilak F (2009). Extreme obesity due to impaired leptin signaling in mice does not cause knee osteoarthritis. Arthritis Rheum.

[5] Nguyen MT, Favelyukis S, Nguyen AK, Reichart D, Scott PA, Jenn A, Liu-Bryan R, Glass CK, Neels JG, Olefsky JM (2007). A subpopulation of macrophages infiltrates hypertrophic adipose tissue and is activated by free fatty acids via toll-like receptors 2 and 4 and JNK-dependent pathways. J Biol Chem.

[6] Bagga D, Wang L, Farias-Eisner R, Glaspy JA, Reddy ST (2003). Differential effects of prostaglandin derived from omega-6 and omega-3 polyunsaturated fatty acids on COX-2 expression and IL-6 secretion. Proc Natl Acad Sci U S A.

[7] Pincus T, Koch G, Lei H, Mangal B, Sokka T, Moskowitz R, Wolfe F, Gibofsky A, Simon L, Zlotnick S (2004). Patient Preference for Placebo, Acetaminophen (paracetamol) or Celecoxib Efficacy Studies (PACES): two randomised, double blind, placebo controlled, crossover clinical trials in patients with knee or hip osteoarthritis. Ann Rheum Dis.

[8] Knott L, Avery NC, Hollander AP, Tarlton JF (2011). Regulation of osteoarthritis by omega-3 (n-3) polyunsaturated fatty acids in a naturally occurring model of disease. Osteoarthritis Cartilage.

[9] Norling LV, Perretti M (2013). The role of omega-3 derived resolvins in arthritis. Curr Opin Pharmacol.

[10] Sarma AV, Powell GL, LaBerge M (2001). Phospholipid composition of articular cartilage boundary lubricant. J Orthop Res.

[11] Kosinska MK, Ludwig TE, Liebisch G, Zhang R, Siebert HC, Wilhelm J, Kaesser U, Dettmeyer RB, Klein H, Ishaque B (2015). Articular joint lubricants during osteoarthritis and rheumatoid arthritis display altered levels and molecular species. PLoS One.

[12] Oh DY, Talukdar S, Bae EJ, Imamura T, Morinaga H, Fan W, Li P, Lu WJ, Watkins SM, Olefsky JM (2010). GPR120 is an omega-3 fatty acid receptor mediating potent anti-inflammatory and insulin-sensitizing effects. Cell.

[13] Ichimura A, Hirasawa A, Poulain-Godefroy O, Bonnefond A, Hara T, Yengo L, Kimura I, Leloire A, Liu N, Iida K (2012). Dysfunction of lipid sensor GPR120 leads to obesity in both mouse and human. Nature.

[14] Zhang D, So WY, Wang Y, Wu SY, Cheng Q, Leung PS (2017). Insulinotropic effects of GPR120 agonists are altered in obese diabetic and obese non-diabetic states. Clin Sci.

[15] Zhang D, Leung PS (2014). Potential roles of GPR120 and its agonists in the management of diabetes. Drug Des Devel Ther.

[16] Quesada-Lopez T, Cereijo R, Turatsinze JV, Planavila A, Cairo M, Gavalda-Navarro A, Peyrou M, Moure R, Iglesias R, Giralt M (2016). The lipid sensor GPR120 promotes brown fat activation and FGF21 release from adipocytes. Nat Commun.

[17] Hayami T, Pickarski M, Zhuo Y, Wesolowski GA, Rodan GA, Duong LT (2006). Characterization of articular cartilage and subchondral bone changes in the rat anterior cruciate ligament transection and meniscectomized models of osteoarthritis. Bone.

[18] Chen Y, Sun Y, Pan X, Ho K, Li G (2015). Joint distraction attenuates osteoarthritis by reducing secondary inflammation, cartilage degeneration and subchondral bone aberrant change. Osteoarthritis Cartilage.

[19] Chen Y, Lin S, Sun Y, Pan X, Xiao L, Zou L, Ho KW, Li G (2016). Translational potential of ginsenoside Rb1 in managing progression of osteoarthritis. J Orthop Translat.

[20] Kilkenny C, Browne WJ, Cuthill IC, Emerson M, Altman DG (2012). Improving bioscience research reporting: the ARRIVE guidelines for reporting animal research. Osteoarthritis Cartilage.

[21] Koren N, Simsa-Maziel S, Shahar R, Schwartz B, Monsonego-Ornan E (2014). Exposure to omega-3 fatty acids at early age accelerate bone growth and improve bone quality. J Nutr Biochem.

[22] Huang MJ, Wang L, Jin DD, Zhang ZM, Chen TY, Jia CH, Wang Y, Zhen XC, Huang B, Yan B (2014). Enhancement of the synthesis of n-3 PUFAs in fat-1 transgenic mice inhibits mTORC1 signalling and delays surgically induced osteoarthritis in comparison with wild-type mice. Ann Rheum Dis.

[23] Wu CL, Jain D, McNeill JN, Little D, Anderson JA, Huebner JL, Kraus VB, Rodriguiz RM, Wetsel WC, Guilak F (2015). Dietary fatty acid content regulates wound repair and the pathogenesis of osteoarthritis following joint injury. Ann Rheum Dis.

[24] Suri S, Walsh DA (2012). Osteochondral alterations in osteoarthritis. Bone.

[25] Hunter DJ, Zhang Y, Niu J, Goggins J, Amin S, LaValley MP, Guermazi A, Genant H, Gale D, Felson DT (2006). Increase in bone marrow lesions associated with cartilage loss: a longitudinal magnetic resonance imaging study of knee osteoarthritis. Arthritis Rheum.

[26] Kim HJ, Yoon HJ, Kim BK, Kang WY, Seong SJ, Lim MS, Kim SY, Yoon YR (2016). G protein-coupled receptor 120 signaling negatively regulates osteoclast differentiation, survival, and function. J Cell Physiol.

[27] Gao B, Huang Q, Jie Q, Lu WG, Wang L, Li XJ, Sun Z, Hu YQ, Chen L, Liu BH (2015). GPR120: a bi-potential mediator to modulate the osteogenic and adipogenic differentiation of BMMSCs. Sci Rep.

[28] Wei L, Fleming BC, Sun X, Teeple E, Wu W, Jay GD, Elsaid KA, Luo J, Machan JT, Chen Q (2010). Comparison of differential biomarkers of osteoarthritis with and without posttraumatic injury in the Hartley guinea pig model. J Orthop Res.

[29] Elsaid KA, Jay GD, Chichester CO (2007). Reduced expression and proteolytic susceptibility of lubricin/superficial zone protein may explain early elevation in the coefficient of friction in the joints of rats with antigen-induced arthritis. Arthritis Rheum.

[30] Kanbe K, Takagishi K, Chen Q (2002). Stimulation of matrix metalloprotease 3 release from human chondrocytes by the interaction of stromal cell-derived factor 1 and CXC chemokine receptor 4. Arthritis Rheum.

[31] Ohashi K, Parker JL, Ouchi N, Higuchi A, Vita JA, Gokce N, Pedersen AA, Kalthoff C, Tullin S, Sams A (2010). Adiponectin promotes macrophage polarization toward an anti-inflammatory phenotype. J Biol Chem.

[32] Wu CL, Kimmerling KA, Little D, Guilak F (2017). Serum and synovial fluid lipidomic profiles predict obesity-associated osteoarthritis, synovitis, and wound repair. Sci Rep.

[33] Trayhurn P, Denyer G (2012). Mining microarray datasets in nutrition: expression of the GPR120 (n-3 fatty acid receptor/sensor) gene is down-regulated in human adipocytes by macrophage secretions. J Nutr Sci.

[34] Rai MF, Hashimoto S, Johnson EE, Janiszak KL, Fitzgerald J, Heber-Katz E, Cheverud JM, Sandell LJ (2012). Heritability of articular cartilage regeneration and its association with ear wound healing in mice. Arthritis Rheum.

[35] Bay-Jensen AC, Reker D, Kjelgaard-Petersen CF, Mobasheri A, Karsdal MA, Ladel C, Henrotin Y, Thudium CS (2016). Osteoarthritis year in review 2015: soluble biomarkers and the BIPED criteria. Osteoarthritis Cartilage.

[36] Bauer DC, Hunter DJ, Abramson SB, Attur M, Corr M, Felson D, Heinegard D, Jordan JM, Kepler TB, Lane NE (2006). Classification of osteoarthritis biomarkers: a proposed approach. Osteoarthritis Cartilage.

[37] Kraus VB, Burnett B, Coindreau J, Cottrell S, Eyre D, Gendreau M, Gardiner J, Garnero P, Hardin J, Henrotin Y (2011). Application of biomarkers in the development of drugs intended for the treatment of osteoarthritis. Osteoarthritis Cartilage.

[38] Thijssen E, van Caam A, van der Kraan PM (2015). Obesity and osteoarthritis, more than just wear and tear: pivotal roles for inflamed adipose tissue and dyslipidaemia in obesity-induced osteoarthritis. Rheumatology.

[39] Berenbaum F (2013). Osteoarthritis as an inflammatory disease (osteoarthritis is not osteoarthrosis!). Osteoarthritis Cartilage.

[40] Daghestani HN, Kraus VB (2015). Inflammatory biomarkers in osteoarthritis. Osteoarthritis Cartilage.

[41] King LK, Henneicke H, Seibel MJ, March L, Anandacoomarasmy A (2015). Association of adipokines and joint biomarkers with cartilage-modifying effects of weight loss in obese subjects. Osteoarthritis Cartilage.

