# Supplementary material for: GPR120 is an important inflammatory regulator in the development of osteoarthritis
Source: Arthritis Res Ther. 2018 Aug 3;20:163. doi: 10.1186/s13075-018-1660-6 (PMC6091098; doi:10.1186/s13075-018-1660-6)
Supplement: Supplementary file 1 — Primer sequence for mouse genotyping. (PDF 52 kb) [file 13075_2018_1660_MOESM1_ESM.pdf]

Additional file 1. Primer sequence for mouse genotyping

Genes	Forward primer	Reverse primer
Gpr120	CGAAAGCATCGCGGAAGAG	GAGTTGGCAAACGTGAAGGC
Neo^r	TTCGGCTATGACTGGGCACAACAG	TACTTTCTCGGCAGGAGCAAGGTG
Gapdh	GCCTTCTCCATGGTGGTGAA	GCACAGTCAAGGCCGAGAAT
