# Supplementary material for: GPR120 is an important inflammatory regulator in the development of osteoarthritis
Source: Arthritis Res Ther. 2018 Aug 3;20:163. doi: 10.1186/s13075-018-1660-6 (PMC6091098; doi:10.1186/s13075-018-1660-6)
Supplement: Supplementary file 2 — Primer sequence for quantitative RT-PCR. (PDF 55 kb) [file 13075_2018_1660_MOESM2_ESM.pdf]

Additional file 2. Primer sequence for Quantitative real-time PCR

Genes	Forward primer	Reverse primer
Human CCL2	GCTCAGCCAGATGCAATCAATG	GTGTCTGGGGAAAGCTAGGG
Human COX2	CAAATTGCTGGCAGGGTTGC	AGGGCTTCAGCATAAAGCGT
Human GAPDH	AACAGCGACACCCACTCCTC	CATACCAGGAAATGAGCTTGACAA
Human GPR120	CCAAAATTTTACAGATCACAAAGGC	CACCACCCAGAAGAAGAGGG
Human IL-1β	AAATACCTGTGGCCTTGGGC	TTTGGGATCTACACTCTCCAGCT
Human MMP13	AAGGAGCATGGCGACTTCT	TGGCCCAGGAGGAAAAGC
