# Supplementary material for: GPR120 is an important inflammatory regulator in the development of osteoarthritis
Source: Arthritis Res Ther. 2018 Aug 3;20:163. doi: 10.1186/s13075-018-1660-6 (PMC6091098; doi:10.1186/s13075-018-1660-6)

Additional file 3. Safranin O and fast green staining of sagittal sections of the subchondral tibia medial compartment in all group. Scale bar, 800 μm.

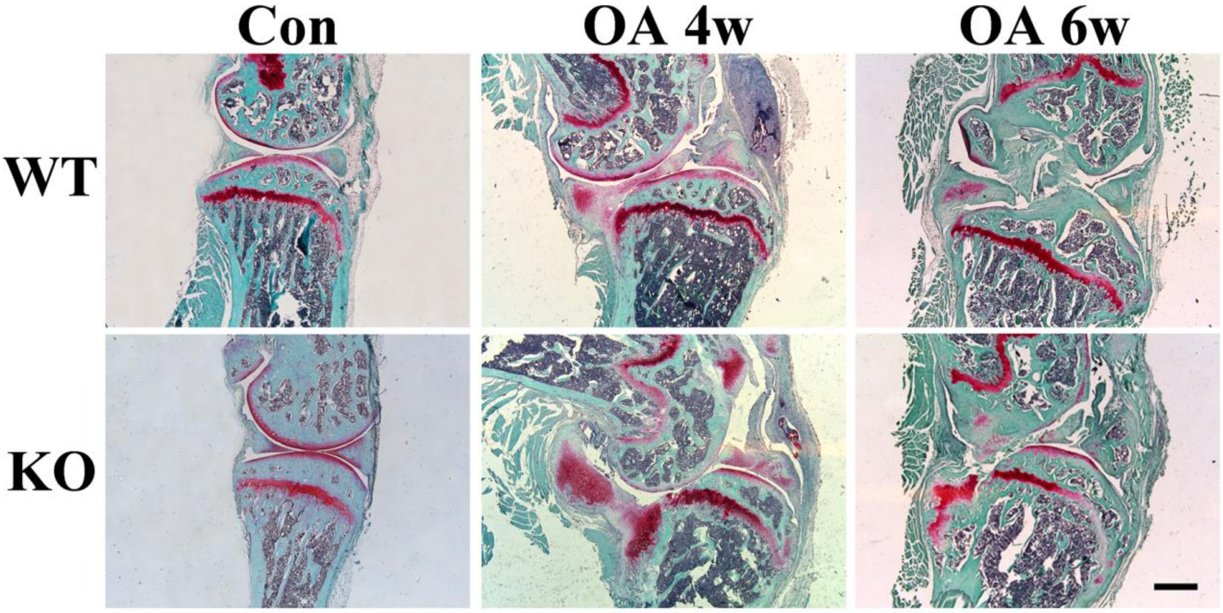

Supplement: Supplementary file 3 — Safranin O and fast green staining of sagittal sections of the subchondral tibia medial compartment in all group. Scale bar = 800 μm. (PDF 230 kb) [file 13075_2018_1660_MOESM3_ESM.pdf]
