# Supplementary material for: GPR120 is an important inflammatory regulator in the development of osteoarthritis
Source: Arthritis Res Ther. 2018 Aug 3;20:163. doi: 10.1186/s13075-018-1660-6 (PMC6091098; doi:10.1186/s13075-018-1660-6)

Additional file.4. Quantitative assay of IHC staining positive cells and structural parameters of subchondral bone by µCT. (A.) percentages of MMP13, (B.) type X collagen (COL X)-positive chondrocytes in articular cartilage and (C.) Osterix positive cells in the tibial subchondral region. n= 5 per group. (D.) Tb.Th. and (E.) SMI. in subchondral bone determined by µCT. n= 10 per group. ***p<0.001, **p<0.01, compare to the WT OA 4 week.

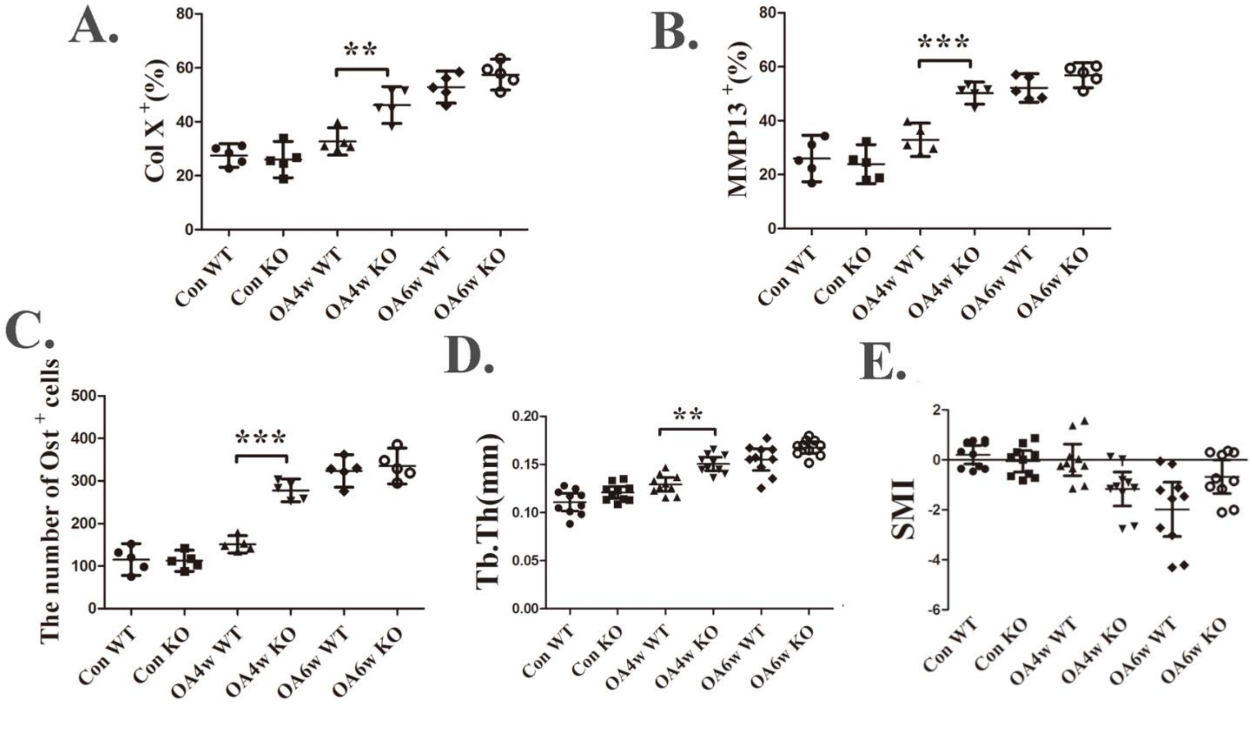

Supplement: Supplementary file 4 — Quantitative assay of immunohistochemical staining for positive cells and structural parameters of subchondral bone by μCT. (A) Percentages of MMP13, (B) type X collagen (COLX)-positive chondrocytes in articular cartilage, and (C) The number of Osterix-positive cells in the tibial subchondral region. (D) Tb.Th. and (E) SMI in subchondral bone determined by μCT. n = 5 per group. ***p < 0.001, **p < 0.01, *p < 0.05, compared with the WT OA at 4 weeks. (PDF 175 kb) [file 13075_2018_1660_MOESM4_ESM.pdf]
