# Supplementary material for: GPR120 is an important inflammatory regulator in the development of osteoarthritis
Source: Arthritis Res Ther. 2018 Aug 3;20:163. doi: 10.1186/s13075-018-1660-6 (PMC6091098; doi:10.1186/s13075-018-1660-6)

Additional file 5. Immunofluorescence staining in human chondrocytes. The result showed that human chondrocytes were type II collagen (Col II)-positive (red). Magnification of the image: ×100.

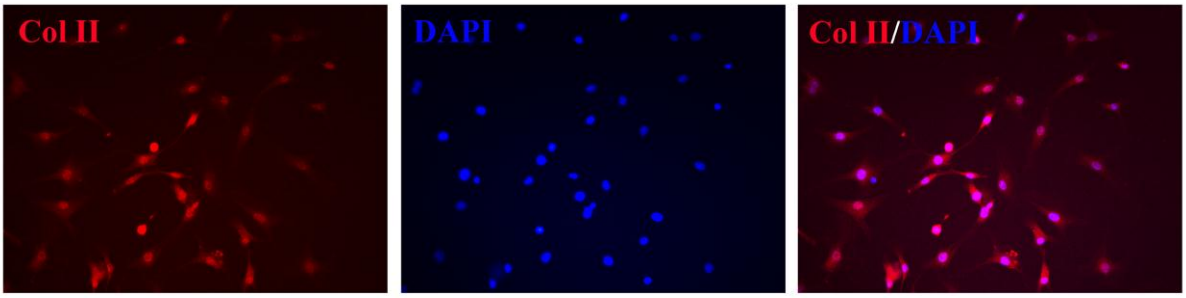

Supplement: Supplementary file 5 — Immunofluorescence staining in human chondrocytes. The result showed that human chondrocytes were type II collagen (Col II)-positive (red). Magnification of the image ×100. (PDF 93 kb) [file 13075_2018_1660_MOESM5_ESM.pdf]
