# Supplementary material for: GPR120 is an important inflammatory regulator in the development of osteoarthritis
Source: Arthritis Res Ther. 2018 Aug 3;20:163. doi: 10.1186/s13075-018-1660-6 (PMC6091098; doi:10.1186/s13075-018-1660-6)

Additional file 6. GPR120 agonist accelerate wound repair in mice skin defected model. (A.) Representative images and quantitative assay of wound area at 8 days post wounding. The DHA treated mice had the smallest wound area as compared to the control mice. Two way ANOVA, * $p < 0.05$. (B.) H&E stained images showed that the mice treated by DHA had a thickened epithelium. The bottom figure are higher-magnification views of the rectangle areas in the upper figure. Black arrow: Epidermis; Red arrow: Hair follicle. Scale bar, 800 µm (in top), 200 µm (in bottom). (C.) Quantitative and (D.) Immunohistochemical analysis of CD68 positive cells (brown) in the wound healing region. The result indicated that the number of macrophage marker (CD68) positive cells in DHA treated mice is down-regulated significantly comparing to the control mice. The bottom figure are higher-magnification views of the rectangle areas in the upper figure. *** $p < 0.001$. Scale bar, 400 µm (in top), 50 µm (in bottom).

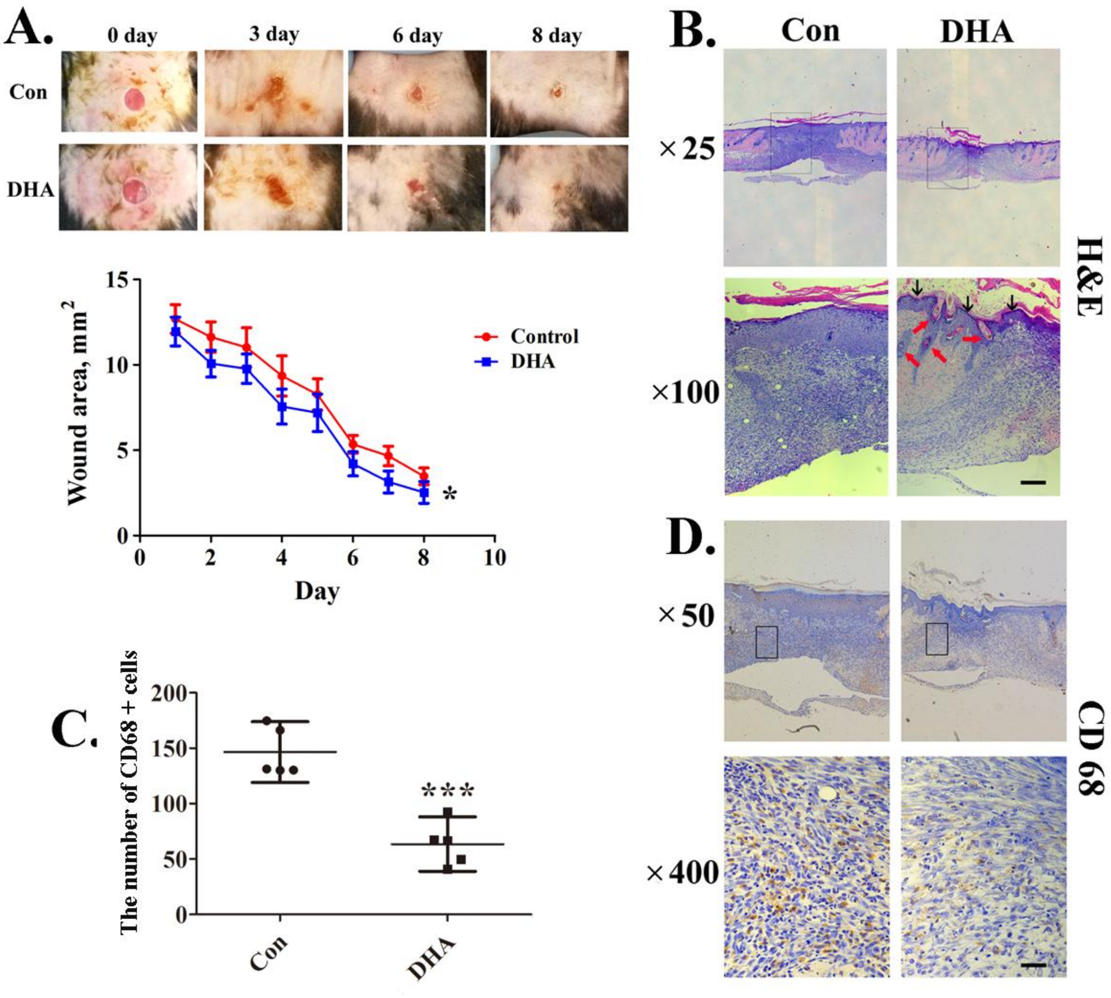

Supplement: Supplementary file 6 — GPR120 agonist accelerates wound repair in a mouse skin defected model. (A) Representative images and quantitative assay of the wound area at 8 days post-wounding. The DHA-treated mice had the smallest wound area compared with the control mice. Two-way ANOVA, *p < 0.05. (B) H&E stained images showed that the mice treated by DHA had a thickened epithelium. The bottom figures are higher magnification views of the rectangle areas in the upper figures. Black arrow: epidermis; red arrow: hair follicle. Scale bar = 800 μm (top), 200 μm (bottom). (C) Quantitative and (D) immunohistochemical analysis of CD68-positive cells (brown) in the wound healing region. The result indicated that the number of macrophage marker (CD68)-positive cells in DHA-treated mice is downregulated significantly compared with the control mice. The bottom figures are higher magnification views of the rectangle areas in the upper figures. ***p < 0.001. Scale bar = 400 μm (top), 50 μm (bottom). (PDF 227 kb) [file 13075_2018_1660_MOESM6_ESM.pdf]
